# Genome-Wide Identification, Classification, and Expression Divergence of Glutathione-Transferase Family in* Brassica rapa* under Multiple Hormone Treatments

**DOI:** 10.1155/2018/6023457

**Published:** 2018-05-24

**Authors:** Nadeem Khan, Chun-mei Hu, Waleed Amjad Khan, Xilin Hou

**Affiliations:** ^1^State Key Laboratory of Crop Genetics and Germplasm Enhancement, Ministry of Science and Technology/College of Horticulture, Nanjing Agricultural University, Nanjing 210095, China; ^2^New Rural Research Institute in Lianyungang, Nanjing Agricultural University, Nanjing 210095, China

## Abstract

The GSTs is one of the most important multifunctional protein families which has been playing a crucial role in the different aspects of plant growth. This extensive study about GSTs may establish a solid foundation for the brief functional analysis of BraGSTs in future. In this study, a total of 75 genes were identified in* B. rapa*. Phylogenetic analysis characterized them into eight different subclasses, while Tau and Phi subclasses were the most numerous. The exon-intron structure and the motif composition of BraGSTs were exhibited accordingly to their subclasses. Notably, we also investigated 15 tandem paralogous pairs of genes, which highlighted that all the pairs were purifying in nature as their synonymous values were lower than 1.00. Duplication analysis indicated that about 45.33% of genes mainly occurred through tandem duplication in* B. rapa*. Predominately, the tandem cluster of genes in subclass Tau was greater than the other subclasses. Furthermore, among eight multiple hormonal treatments (ABA, GA, BR, ETH, IAA, IBA, NPA, and JA), most number of BraGSTs was activated by NPA, BR, and ABA treatments. This analysis has provided comprehensive information about GSTs family which may assist in elucidating their exact functions in* B. rapa*.

## 1. Introduction

The plant glutathione transferases, formally glutathione-s-transferase (GSTs; EC 2.5.1.18), are known for their diversity, being multifunctional proteins, and being widely distributed in most of the organisms. To date, the role of GST proteins was considered to be crucial in multiple plant functions such as herbicide detoxification, plant developmental processes, signal transduction, oxidative damage, heavy metals toxicity, and other several key biotic and abiotic factors [1, 2]. It has been reported that GSTs participated in the endogenous developmental process, for example, in maize [3], petunia [4],* Arabidopsis* [5], grapevine, and cyclamen [6]; they were involved in flavonoid binding. The versatility of the GSTs was further extended through previous studies, which have proved their involvement in the regulation of genes. It increases the transcript level through exposure to biotic and abiotic factors including hormones, specifically auxins, ethylene, salicylic acid (SA), abscisic acid (ABA), and methyl jasmonate (MeJA) [7, 8].

The GSTs superfamily have shown variation in their structure and sequences, although they share structural homology based on considered codomain thioredoxin/glutaredoxin-like N-terminal and a larger C-terminal. Thus, variability in nature leads to different hydrophobic substrate specificities in plant GSTs [9]. On the other hand, as compared to mammals plant, GSTs possess a larger cleft for co-substrate, as a result it accepted much more and larger diverse substrates [10, 11]. The GSTs can be categorized into eight different subclasses based on sequence similarities of amino acid and genes structure such as Phi, Tau, Lambda, Zeta, Theta, Lambda, dehydroascorbate reductase (DHAR), *γ*-subunit of translation elongation factor (EF1G), and tetrachlorohydroquinone dehalogenase (TCHQD). Subclasses of GSTs like Phi, Tau, Lambda, and DHAR are considered as plant-specific [12] while Phi and Tau are the most numerous and highly functioning subclasses for their involvement in the detoxification of xenobiotics [13]. Predominantly, most of the GSTs were characterized and investigated in several plant species belonging to Phi and Tau subclass. The overexpression of different genes member of Phi and Tau subclass has been reported to increase the herbicide tolerance [14]; salinity and oxidative stress [15, 16]; and chilling stress [17]. Meanwhile, in Sorghum, 62.5% of genes from subclass Tau have shown significant responses against multiple abiotic factors such as cold, drought, and salinity stresses [7]. However, others subclasses of GSTs such as Lambda and DHAR are mainly involved in redox and thiol transfer reactions rather than xenobiotics [18, 19]. DHAR subclass plays important role in stress resistance and is involved in the ascorbate-GSH recycling reactions [20–22]. Although, two subclasses theta and zeta mainly helps in the function as GSH-dependent peroxidase, isomerase, and act as counterparts in the mammalian system [23, 24]. The EFIG class is basically based on two domains, a typical GST and EF1G domain, which mainly function as GSH peroxidase [25]. GSTs stimulate the scavenging pathways and help them to reduce the effect of toxic material in plant tissues [26]. In addition, glutathione is a specific tripeptide, produced by the combination of glutamic acid, cysteine, and glycine, and referred to as gamma-glutamyl-cysteinylglycine [27]. It is present in body fluid (blood) and cells [28]. On the other hand, it is also located in cytosol and other cell organelles such as endoplasmic reticulum, nucleus, and mitochondria [29, 30]. In particular, glutathione plays a key role in various metabolic pathways that focused on improving the quality of nutrients. Its functions are also linked with the controlling the gene expression patterns, signal transduction, synthesis of DNA, and protein [28]. It has been reported recently that allyl isothiocyanate elevated the expression of* GST* genes by inducing the oxidative stress in* A. thaliana *[31]. The above studies have provided a brief background of GSTs and their importance in plant cell structure and function. However, their associated endogenous role has still need to be identified in* B. rapa,* against such multiple hormone treatments, and thus is largely obscure.

The* B. rapa* genome (Chiifu-401-42) has recently been sequenced and assembled [32], which elucidated its close relationships with* A. thaliana* and after its divergence, it has experienced a whole genome triplication event (WGT) [33, 34]. In this study, a total of 75 GSTs family members were identified in* B. rapa *genome. Here, we conducted a systematic study of* GST* genes family in* B. rapa*, to identify the characterization and phylogenetic relationships with* A. thaliana* and rice along with collinear correlation between* B. rapa* and* A. thaliana*. Interaction networks and expression divergence of BraGSTs under multiple hormonal treatments were also investigated. As a result, this study provides a foundation in understanding the mechanism of GSTs family in* B. rapa* and valuable information for further investigation of hormonal stress responsive genes.

## 2. Materials and Methods

### 2.1. Identification of the GST Genes in* Brassica rapa*

From the Brassica database (BRAD; http://brassicadb.org/brad/) [34], all the genome sequence datasets were downloaded. The* A. thaliana *GST sequences were retrieved from the Arabidopsis Information Resource database (http://www.arabidopsis.org/). The gene information of rice were obtained from http://rice.plantbiology.msu.edu/ [35], based on the previous reported study [36]. The* A. thaliana* and rice were used as queries by performing BLASTP search to identify the putative GST proteins with best domain* e*-value cut-off 1.0. For validation, these potential sequences SMART (http://smart.embl-heidelberg.de/) and the National Center for Biotechnology Information (NCBI) database (http://www.ncbi.nlm.nih.gov/) sever were used. Finally, the physicochemical properties of the BraGSTs were analyzed through Expasy protparam (http://web.expasy.org/protparam/) and the subcellular localization was predicted by WoLF PSORT (https://www.genscript.com/wolf-psort.html).

### 2.2. Phylogenetic Analysis and Genomic Organizations Prediction

The full length protein sequences of BraGSTs were aligned through MUSCLE with default parameters [37]. For each analysis, Maximum Likelihood Method was used for constructing phylogenetic tree with JTT model (Jones-Taylor and Thornton amino acid substitution), using MEGA 7 [38]. The nucleotide divergence for all the GSTs was also analyzed through MEGA 7 with Jukes-Cantor model (1000 bootstrap values).

The coding sequences and the corresponding genomic sequences of BraGSTs were predicted by online tool (http://gsds.cbi.pku.edu.cn/) [39], for exon-intron structure. For the identification of conserved motifs of the GSTs in* B. rapa*, MEME (Multiple Expectation-Maximization for Motif Elicitation program) online server was used (Program version 4.12.0) [39]. The following parameters were implemented, maximum number of motifs 12 and optimum motif widths 12 and 120, while other parameters were set as default.

### 2.3. *Ks* and *Ka* Calculation

The synonymous (*Ks*) and nonsynonymous (*Ka*) substitution rates among the tandem pairs of* BraGST* genes were calculated with the help of MEGA 7 software, based on the coding sequences alignment following the Nei and Gojobori model implemented in MEGA 7 [38].

Additionally, the divergence time was calculated through the following formula: *T* = *Ks*/2*r* in which *r* was taken 1.5 × 10^−8^ (synonymous substitution/year) by showing the rate of divergence [40].

### 2.4. Promoter Analysis and Proteins Interaction Network Prediction

We explored a GFF (generic file format) from the* B. rapa* genome for all the GST promoter sequences (15,00 bp upstream). For each gene, we identified the cis-regulatory element by Plant CARE database (http://bioinformatics.psb.ugent.be/webtools/plantcare/html/). Additionally, with the help of STRING software (https://string-db.org/), we constructed an interaction network among* BraGST* proteins.

### 2.5. Chromosomal Localization and the Syntenic Paralog Pairs of BraGSTs

All the* BraGST* genes were mapped on the ten* B. rapa* chromosomes based on their respective position in genome annotation with the random distribution patterns. The images for all the BraGSTs were drawn through Mapchart [41]. The syntenic relationship between* A. thaliana* and* B. rapa* was explored by searching the term “syntenic genes” in the* B. rapa* database [42]. To present the syntenic relationship on their chromosomes, Circos program [43] was applied.

### 2.6. Pearson Correlation (PCC) Analysis

The PCC values for RNA-seq data and expression patterns among the tandem pairs of BraGSTs were performed on excel sheet 2013.

### 2.7. RNA Isolation and Expression Pattern Analysis for BraGSTs in Five Tissues

The total RNAs were isolated from young leaves using an RNA kit (TaKaRa, Dalian, China). The RNAs were reverse-transcribed into cDNA with a PrimeScript RT reagent kit (TaKaRa, Dalian, China). For qRT-PCR, gene specific primers were designed through Becan designer 7 software and for internal control the* B. rapa* actin gene (Bra028615) was used (Supplementary, Table 1). Step one plus real-time PCR system (Applied Biosystems, Carlsbad, CA) was used for the reactions. The following PCR parameters were used: 94°C for 30 s, 40 cycles at 94°C for 05 s, 60°C for 15 s, and 72°C for 10 s, following by melting curve analysis (61 cycles at 65°C for 10 s). The relative gene expressions were further calculated by the comparative Ct value method [44]. For expression analysis of GSTs in* B. rapa*, we utilized the five accession of Chifu-401-42 (root, stem, leaf, flower, and silique) and the RNA-seq data were generated from previous reports [32]. Hence, the gene expression values were analyzed for each tissue and the fragments per kilobase of transcript per Million fragments mapped (FPKM) were quantified first and then heat maps were generated by using online tools (http://www.omicshare.com/).

### 2.8. Plant Materials and Hormone Treatments

The typical Chinese cabbage cultivar Chiffu-401-42 was used in our experiment, as this cultivar being prominently used for research studies due to the completion of its whole genome-sequencing. Seeds were first treated with sodium hypochlorite (14%) and then were raised on 0.5 MS agar plates (0.7%) in dark for three-day interval at 23°C. After that, the germinated seeds were raised in a controlled environment using small pots with 3 : 1 (soil : vermiculite mixture); growth chamber was programmed with temperature of 27°C; for photoperiod, the duration of light was 16 h and dark 8 h with 60% relative humidity in the greenhouse of Nanjing Agricultural University. One-month old seedlings were transplanted at five leaf-stage into 1/2 Hoagland's solution in small plastic pots with a pH at 6.5. After ten days of acclimatization, seedling of Chinese cabbage was grown with following 8 multiple treatments: (1) ABA 100 *μ*M; (2) GA 100 *μ*M; (3) BR 50 *μ*M; (4) ETH 50 *μ*M; (5) IAA 80 *μ*M; (6) IBA 80 *μ*M; (7) NPA 100 *μ*M; (8) JA 100 *μ*M. We sampled at continuous intervals of 1, 6, and 12 h with three biological triplicates for each sample. The young leaf samples were immediately frozen in liquid nitrogen and stored at −70°C for further analysis.

## 3. Results

### 3.1. Identification of GST Proteins and Their Classification Pattern

**Figure 1:**
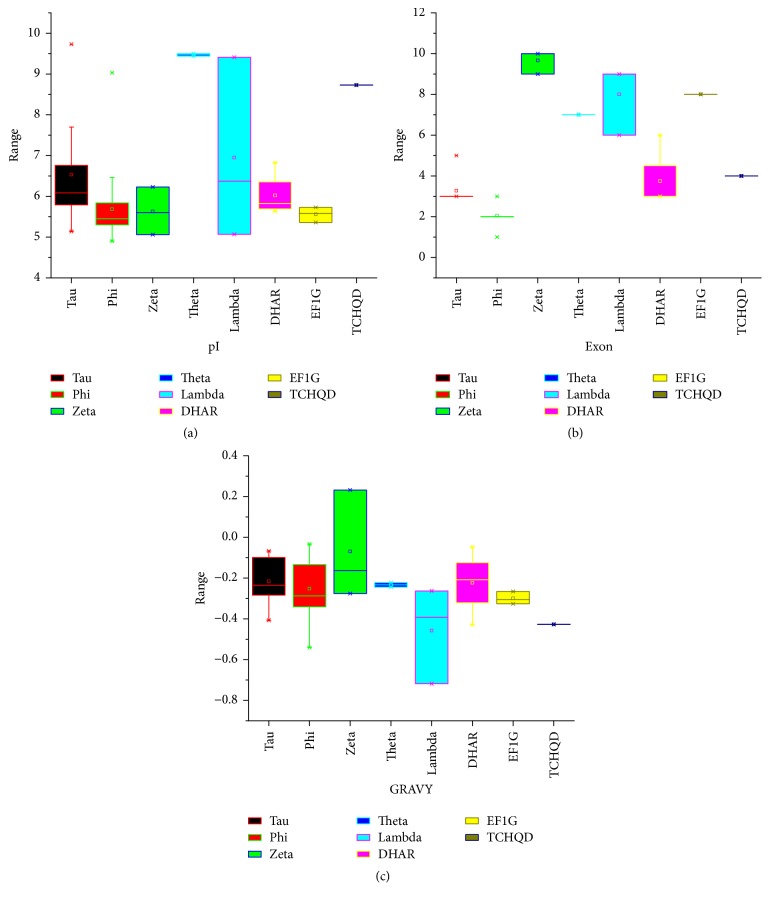
(a) Indicating the pI values among different subclasses of BraGSTs. (b) The number of exons among subclasses of BraGSTs. (c) Showing the grand average of hydropathicity (GRAVY) among subclasses of BraGSTs.

We identified all the putative* GST* genes in* B. rapa* through systemic BLASTP search against the* B. rapa* database, using the query sequences of* A. thaliana* (55) and rice (77). This search resulted in the identification of 75 GST proteins. Subsequently, all these protein sequences were subjected to SMART and NCBI for further verification analyses, and both GST N- and C-terminal domains were confirmed in* B. rapa *genome. To reveal the subclasses of GSTs in* B. rapa,* the results were based on conserved domains in the context of proposed nomenclature for GSTs [45, 46]; it can be further divided into eight different subgroups as Phi, Tau, Zeta, Theta, Lambda, DHAR, EF1G, and TCHQD. The BraGSTs according to the subclasses were designated as* BraGST*. The genes of subclasses were named as BraGSTF, BraGSTU, BraGSTZ, BraGSTT, BraGSTL, BraDHAR, BraEF1G, and BraTCHQD, respectively, and corresponding number for each gene was, for example, BraGSTF1. From the predicted protein sequences of all the BraGSTs, we have collected all the basic description about their length, molecular weight (MW), isoelectric points (pI), grand average of hydropathicity (GRAVY), exon number, aliphatic index, subcellular prediction, and others which are briefly described (Supplementary, Table 2). In general, the amino acids for all the BraGSTs were varied 167–566 and the MW ranged 18.85–64.24 kDa, while the highest MW and length were recorded for subclass of EFIG with an average of 52.37 kDa and 464. The pI values were ranged 4.9–9.73, which speculated that different BraGST proteins may function in different microenvironments as described in Figure 1(a) and Supplementary Table 2, while the number of exons ranged 1–10 for all the subclasses of BraGSTs (Figure 1(b) and Supplementary Table 2). Though, the GRAVY from the subclass of BraGSTZ with only one protein were hydrophobic in nature, whereas the rest of them showed hydrophilic properties (Figure 1(c) and Supplementary, Table 2). To understand plant functions, protein localization is particularly important. In our study, most of the subclasses of BraGSTs were predicted to be located in mitochondria, chloroplast, and cytoplasm, while only a small number of proteins were involved in plasma membrane, vacuole, and nucleus.

### 3.2. Expansion and Structural Characteristics of BraGST Genes in* Brassica rapa*

**Figure 2:**
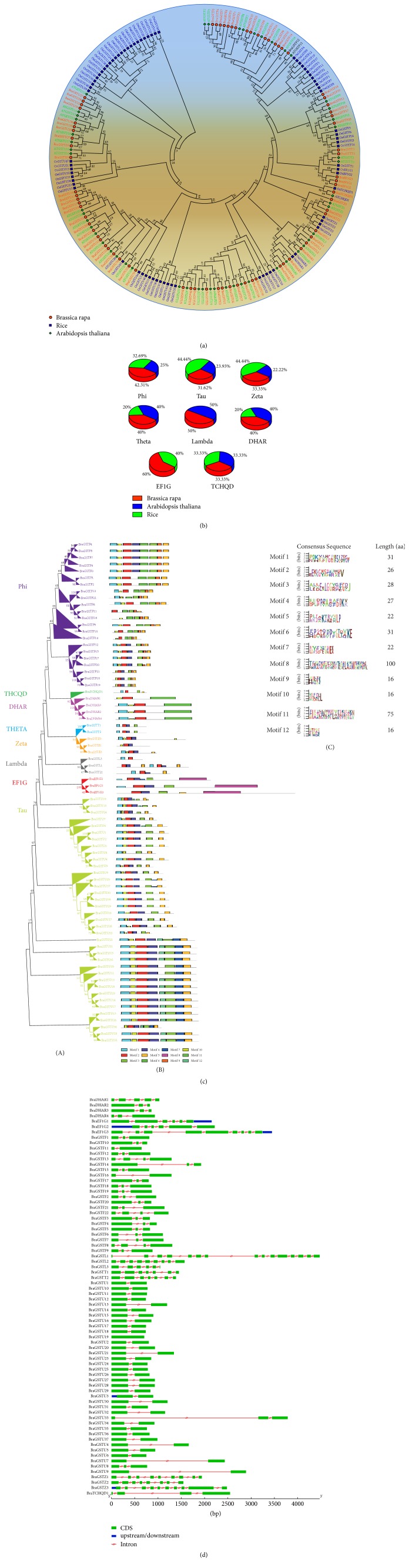
(a) Phylogenetic relationship of BraGSTs among three species* B. rapa, A. thaliana*, and rice. The phylogenetic tree was constructed by MEGA 7 using the Maximum Likelihood Method (1000 bootstraps). Genes of different species are marked with different colors. (b) Relative classification patterns of BraGSTs family based on number of genes between subclasses of BraGSTs among three species. (c) Phylogenetic tree, motif structure, and LOGO. (A) The phylogenetic tree was constructed by MEGA 7 using the Maximum Likelihood Method (1000 bootstraps). (B) The conserved motif of BraGSTs was elucidated by MEME. Different motifs and their positions are represented by different colors, respectively, numbered 1–12 at the bottom. (C) The consensus sequence of conserved motifs of BraGSTs and predicted length (amino acids) for each motif are given. (d) The exon-intron and upstream/downstream region are represented by green boxes, red line, and blue boxes, respectively. At the bottom of the figure the relative position is proportionally displayed based on the kilobase scale.

To validate the* GST* genes relationship among* B. rapa, A. thaliana*, and rice, all the putative sequences were aligned with MUSCLE in MEGA 7, to generate unrooted tree with Maximum Likelihood Method (Figure 2(a)). All the classes belonging to the eight groups of BraGSTs were in clusters with their counterparts of* A. thaliana* and rice. It also verified the reliability of our classification of BraGSTs, based on the conserved domain with completely matched results. The classification patterns of number* BraGST* genes for each subclasses are described in Table 1, while, for the comparative analysis with* A. thaliana* and rice, the results are shown in Figure 2(b). We also presented a phylogenetic tree with all the* BraGST *genes to verify the extent and lineage-expansion; eight subclasses were marked with different color (Figure 2(c)). As described in Figure 2(c), subclass Tau (BraGSTU) has contained the largest (37) number of genes followed by Phi (22). Our results were in accordance with previously reported studies [7, 36, 47]. We also investigated the sequence features of BraGSTs through MEME program, which are used for predicting the conserved motifs; at least 12 motifs were identified and named as motifs 1–12. Through MEME, we also obtained the LOGO of these motifs and presented them along with their motifs (Figure 2(c)). The BraGSTs were mainly distributed with similar patterns of motifs according to the eight subclasses. However, motifs 1-2 were found almost among all the BraGSTs, suggesting their highly conserved domain. Besides the common patterns, subclass EFIG has contained higher number of motifs than any other subclass of BraGSTs. Noticeably, motif 8 was found to have higher number (88) of consensus sequences while motifs 9, 10, and 12 contained fewer (16) number of consensus sequences. In addition, we also compared the genomic and cDNA sequences; we found that all the subclasses showed higher than one intron, while specifically subclass Lambda was found to contain the high (14) number of intron (Figure 2(d)). However, the structure of Lambda was varied in nature in other genes, while DHAR and Phi showed uniformity in their structure. Notably, the gene structure of all the BraGSTs was consistent according to the subclasses.

### 3.3. Copy Number Variation/Gene Retention and Collinearity Analysis of BraGSTs

**Figure 3:**
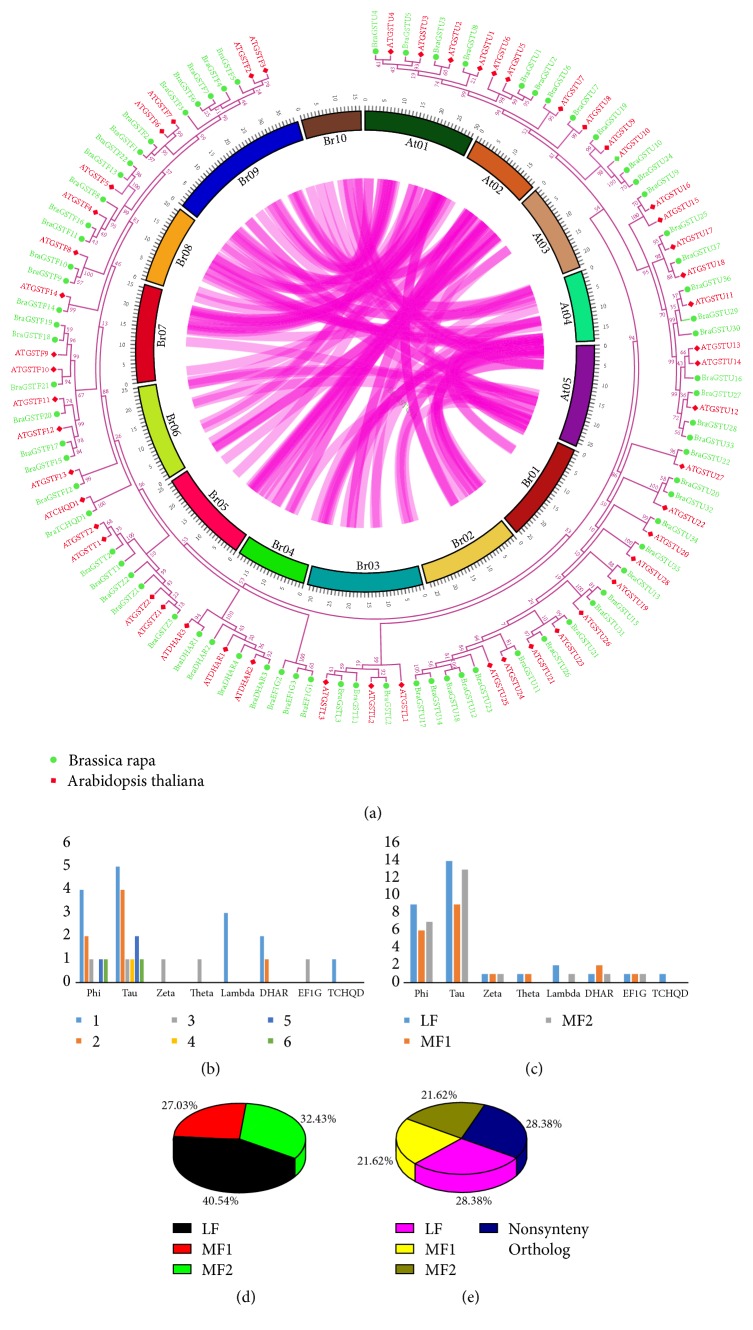
(a) The collinear correlation for all the genes of BraGST was displayed between* B. rapa *and* A. thaliana.* The ten Chinese cabbage chromosomes (Br01–Br10) and the five* A. thaliana *chromosomes (At1–At5) are shown in different random colors. The illustration was drawn using Circos software. (b) Showing copy number of variation among different subclasses of BraGSTs. (c) Showing gene retention among three subgenomes of BraGSTs in* Brassica rapa*. (d) Showing the ratio of* BraGST* genes among three subgenomes. (e) Showing the ratio of* BraGST *genes among three subgenomes along with nonsynteny ortholog.

To determine the copy number of variation during specific whole genome triplication event (WGT), we investigated the* BraGST* genes in* A. thaliana* and* B. rapa *and the collinear relationships are shown in (Figure 3(a)). Surprisingly, we found up to six numbers of copy variation between two different subclasses of BraGSTs, i.e., Phi and Tau, due to high tandem array of genes (Figure 3(b)). In subclass Tau, we explored five and four copies of genes, whereas single copy of genes was also found in subclass Tau. Our findings also demonstrated different retention of genes in the subclasses of BraGSTs, such as Phi 21/21, Tau 36/38, Zeta 3/3, Theta 2/2, Lambda 3/3, DHAR 4/4, EF1G 3/3, and TCHQD 1/1, respectively, and these results revealed high number of retention genes among all the subclasses of BraGSTs (Supplementary Table 3 and Figure 3(c)). The* B. rapa* contains three genomes, as based on the degree of fractionation, namely, least fractionation (LF), medium fractionated (MF1), and most fractionated (MF2). During this study, we presented the ratio of all the BraGSTs in the three subgenomes, LF genome showed the highest 40.54% of genes, and noticeably MF2 showed 32.43%, which were higher than MF1 27.03% (Figure 3(d)). On the other hand, we also highlighted the ratio of these three subgenomes with nonsynteny genes of BraGSTs; interestingly the nonsynteny ortholog and LF subgenome share 28.38% each and MF1 and MF2 share an equal of 21.62% as shown in (Figure 3(e)).

### 3.4. Chromosomal Localization and Tandem Array Selective Pressure Analysis of the BraGSTs in* B. rapa*

**Figure 4:**
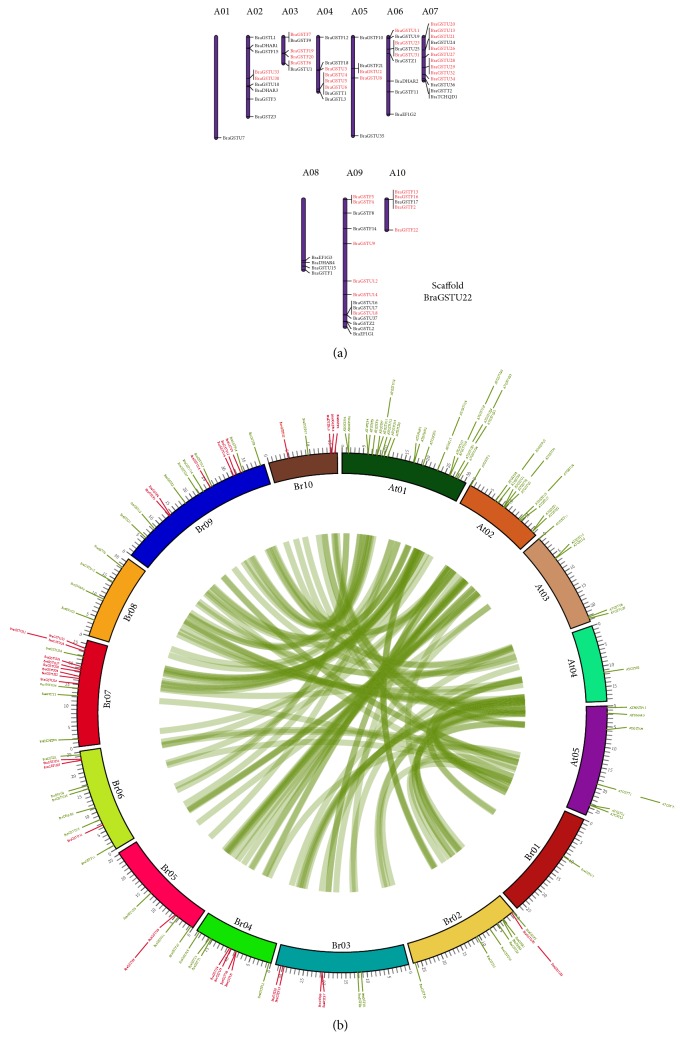
(a) Chromosome location of the BraGSTs was obtained from the GFF file and displayed by using Mapchart. The duplication type tandem array was displayed with red color. (b) Syntenic relationship between* B. rapa* and* A. thaliana *was displayed through Circos program, while tandem array was marked with red color.

All the BraGSTs were positioned on the ten* B. rapa *chromosomes (A01–A10) with a random distribution. Chromosome A09 contained the most* BraGST* genes (18.92%), followed by A07 with (17.57%), whereas chromosome A01 contained the fewest genes (1.35%). Furthermore, the duplication type (tandem array) was identified through MCScanX program; about 34 genes were identified with a random distribution on A01–A10 chromosomes and were marked with red color. Specifically, we observed that most of the tandem arrays were clustered in the region of chromosome. For example, 9 tandem array genes were clustered on A07 chromosome, all of them belonged to subclass Tau, and the only gene which was on scaffold also belonged to subclass Tau (Figure 4(a)). Additionally, we also presented a relationship of the syntenic region of BraGSTs along with* A. thaliana* using Circos software and the tandem array of* BraGST* genes was marked with red color (Figure 4(b)).

A total of 15 pairs of tandem array were analyzed for selective pressure using Mega 7 and the divergence time of the duplicated genes was estimated by calculating the number of synonymous substitutions (*Ks*) and nonsynonymous substitution rates (*Ka*). All of the tandem pairs showed less than 1.00*Ka*/*Ks* ratio, which suggest the purifying selection of the genes. The values were varied 0.13–0.77 with an average divergence of 29.80 MYA (Table 2). These results speculated that the slow evolving nature of the tandem array of BraGSTs with a variation patterns in plant functions. The purifying nature of BraGSTs suggested the maintenance function in* B. rapa.*

### 3.5. Expression Pattern of BraGSTs in Different Tissues and the Corelation Networking Analysis of Tandem Pairs

**Figure 5:**
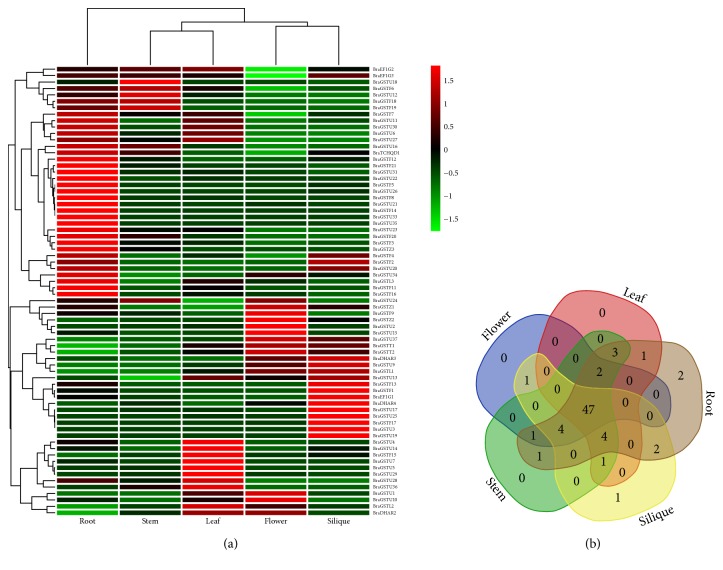
(a) Heatmap of expression profiles (in log* *2-based FPKM) for BraGSTs in the five tissues of stem, root, leaf, flower, and silique. The expression levels are exhibited by the color bar. (b) Venn diagram analysis of the tissue-expression of BraGSTs.

To investigate putative roles of* BraGST* genes in* B. rapa* growth and development, we analyzed the expression patterns across five tissues (roots, stems, leaves, flowers, and siliques) using RNA-seq data [32]. Most of the BraGSTs demonstrated a high alteration in expression level in tissues-specific patterns as shown in (Figure 5(a)). For example, BraGTF18 and BraGSTF19, belonging to subclass Phi, exhibited higher expression in stem and root as compared to any other tissues, indicating that they may function in stem and root development. Meanwhile, some of the genes like from subclasses Phi, Tau, and DHAR such as BraGSTF10 and BraGST22; BraGSTU8 and BraGSTU32; and BraDHAR1 showed no expression, while BraGST11, BraGSTF17, BraGSTU3, BraGSTU19, BraGSTU33, and GSTZ3 have slight expression in any tissue (Figure 5(a) and Supplementary Table 4). We also presented tissue-specific genes; interestingly 2 genes were found in root and 1 was in silique, which highlighted the tissue-specific developmental role (Figure 5(b)).

**Figure 6:**
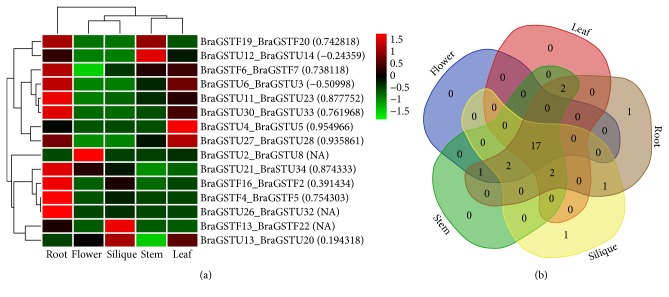
(a) Heatmap of expression profiles for BraGSTs 15 tandem paralogous pairs in the five tissues of stem, root, leaf, flower, and silique. The Pearson correlation coefficients (PCC) are also displayed in bracket while NA indicates no available results for PCC. (b) Venn diagram analysis of the tissue-expression of BraGSTs.

We also investigated trends of expression patterns among 15 pairs of tandem array and their Pearson correlation. Notably, our results demonstrated that most of the tandem pairs exhibited a high expression pattern across five tissues. Intriguingly, two tandem pairs from subclass of Phi, such as (BraGSTF19_ BraGSTF20), showed a higher expression peak in root and stem, which could be speculated that these pairs due to the similarity in high expression may be involved on the same pathway for root and stem development. Meanwhile, the correlation between these two pairs was recorded (0.742818), which also shed light on their close relationship (Figure 6(a) and Supplementary Table 5). However, two pairs from subclasses of Phi and Tau were detected (BraGSTF13_BraGSTF22 and BraGSTU33_BraGSTU33), showing selective expression in specific tissues. Moreover, most of the pairs showed a high correlation among each other, while two pairs were found with negative values like BraGSTU6_ BraGSTU3 and BraGSTU12_ BraGSTU14 and three pairs had no PCC values, the reason for that is these pairs might lose function by pseudogenization. In tissue-specific clustering, we observed only one specific gene located in root and silique each, suggesting their possible role in function of root and stem development (Figure 6(b)).

**Figure 7:**
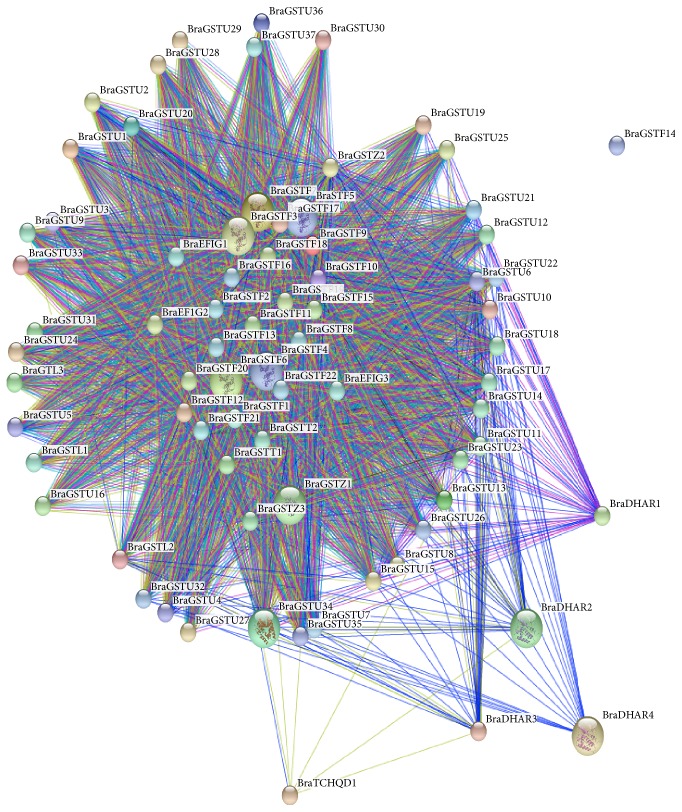
The coregulatory network for BraGSTs was presented by STRING sever.

To elucidate the coregulatory network among BraGSTs, we presented a protein-protein interaction structure, in which their functional and physical properties were examined by using STRING server (Figure 7). Most of the BraGSTs showed a highly interacting network with other proteins, except BraGSTF14. The protein interaction is not necessary to have a similar relation among the same subclasses but can also exist among different subclasses of a gene family. Predominately, most of the proteins were located in the center of the network were Phi, Zeta, Theta, and EF1G, suggesting that these proteins with other BraGSTs have a more complex interaction relationship.

### 3.6. Expression Profiling and Coregulatory Networks of BraGST Genes in Response to Multiple Hormone Treatments

**Figure 8:**
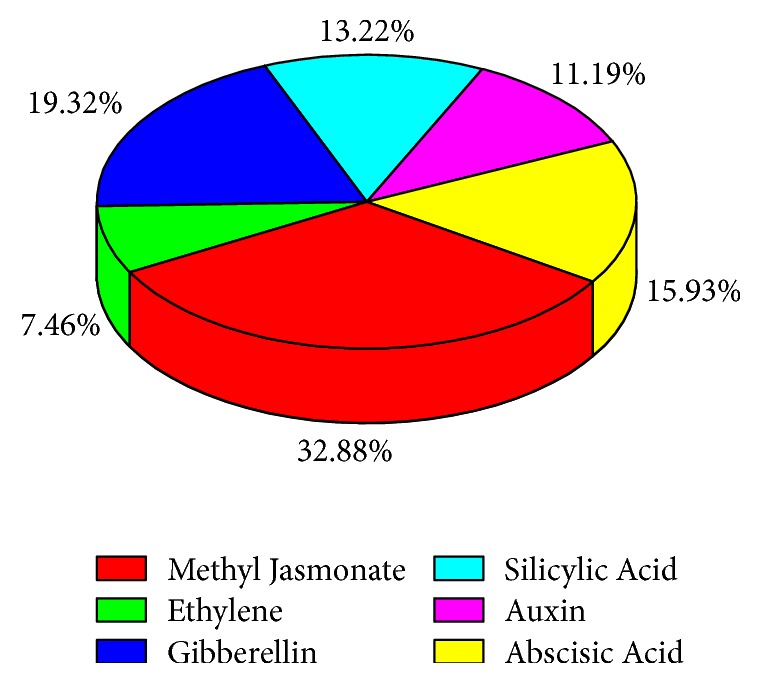
Showing the ratio of different cis-element.

During recent times, many researchers primarily are focusing on how to understand plant functions under various stimulation. GSTs are to be involved in various biotic, abiotic, and hormone stresses such as auxins, ABA, and ethylene as reported in previous studies [48]. Keeping in view the importance of GSTs, we explored PlantCARE (plant cis-acting regulatory element database) for the identification of motifs only existing for hormonal stresses (Supplementary Table 6). The results demonstrated that most of the* BraGST* genes were involved in methyl jasmonate (32.88%), gibberellin (19.32%), abscisic acid (15.93%), salicylic acid (13.22%), and auxin (11.19%) and fewest genes were found in ethylene (7.46%) (Figure 8). Consequently, these results speculated that MeJA, GA, ABA, SA, auxin, and ethylene could affect the expression level of BraGSTs. For the functional dissection of GSTs under response to hormone stresses, mainly cis-element provided indirect evidences. However, little is known about its function in* B. rapa* response to hormonal stresses. To access the changes and the diversity of BraGSTs, further experimental validation step is required in future.

**Figure 9:**
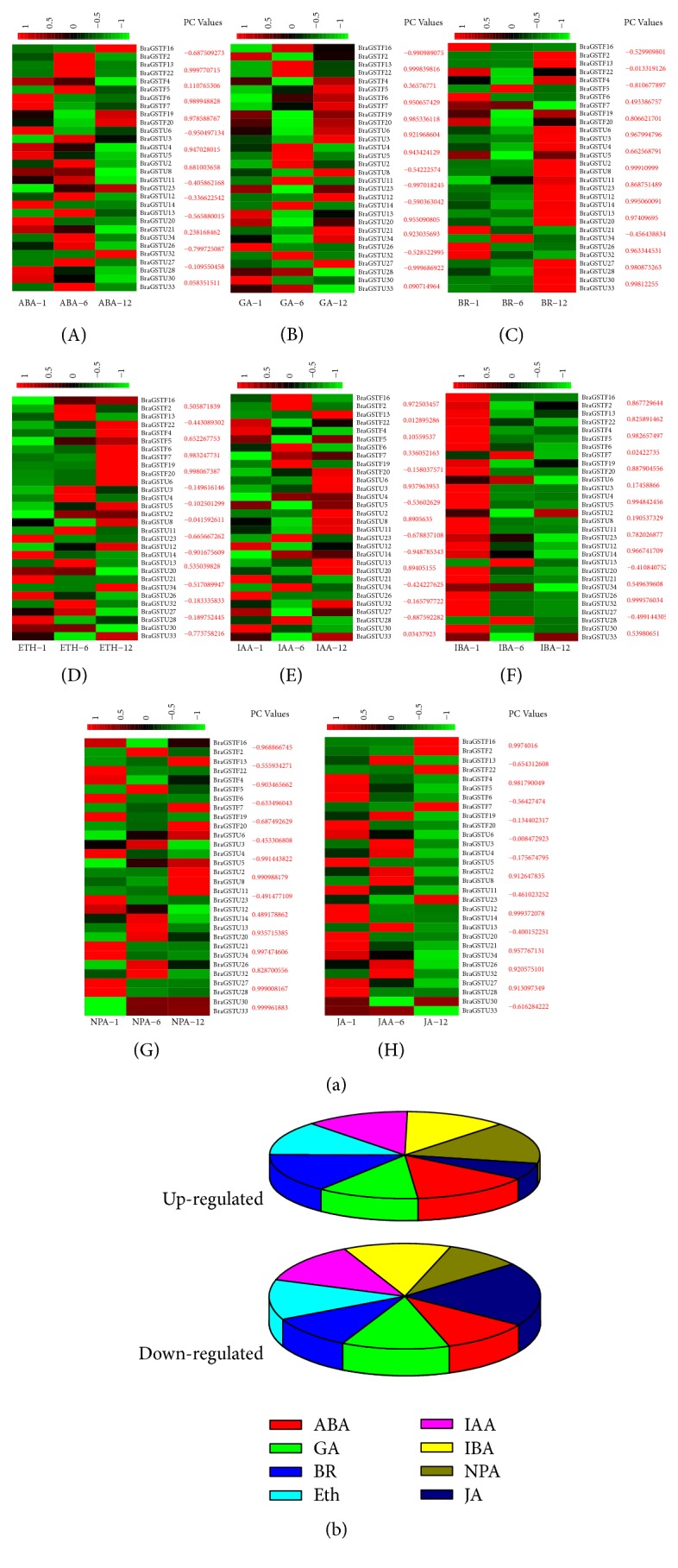
(a) Expression analysis of the* BraGST* genes under eight multiple hormonal treatments in* B. rapa *(A–H). Heatmap representation of the* BraGST *genes under eight multiple treatments, namely, ABA, GA, BR, ETH, IAA, IBA, NPA, and JA. For each pair of BraGSTs their PCC values are also displayed. (b) Showing the up- and downregulated genes in response to multiple treatments.

**Figure 10:**
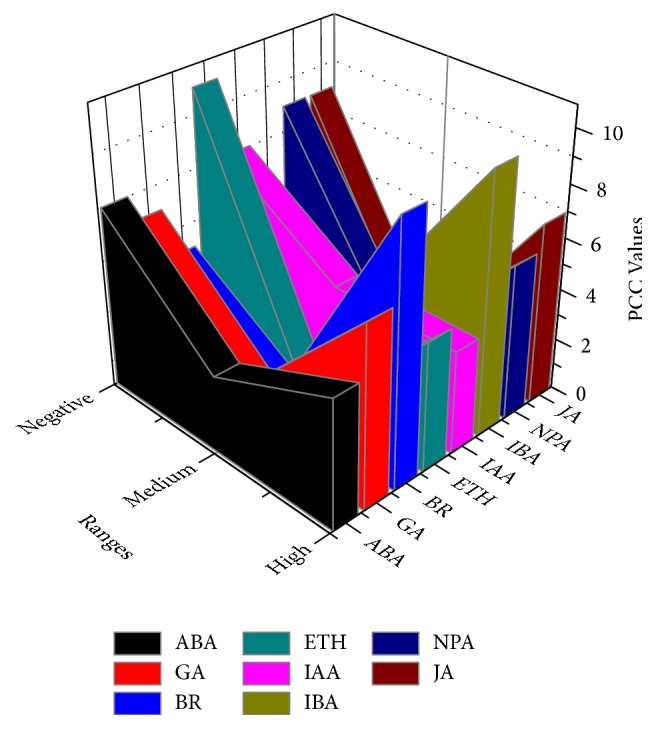
Showing the PCC values under response to multiple hormone treatments.

To understand the expression profiles of* BraGST* genes under eight different hormonal treatments, the expression patterns for 15 paralogous pairs of tandem array were studied using qRT-PCR experiment. Heatmap was generated in response to multiple hormone treatments for transcript expression fold change as shown in (Figure 9(a) and Supplementary Table 7). Most of the BraGSTs showed a high striking expression patterns during various hormone treatments (namely, ABA, GA, BR, ETH, IAA, IBA, NPA, and JA). More genes were particularly induced by NPA than any other hormones and expressed a higher proportion of upregulation (66%) and relatively lower downregulated level (34%), followed by BR (61%) and (39%), ABA (60%), and (40%). Meanwhile, JA were found to be sensitive to treatment as most of the genes were downregulated up to 74% and only 24% were upregulated (Figure 9(b)). To understand the correlation and coregulatory network among the tandem pairs, we also calculated the PCC values based on their relative expression values. For the correlation network, we have arranged them into three categories with respect to PCC values, such as greater than 0.6 (High), less than 0.5 but greater than 0 (Medium), and negative values with (Negative) correlation (Figure 10(a) and Supplementary Table 8). BR and IBA showed a higher and closer relationship among each other with 10 PCC values each, while ETH were found with a high number of 10 negative PCC values, suggesting its contrasting nature among the tandem pairs of genes.

## 4. Discussion

### 4.1. Identification, Phylogeny, and Gene Duplication of BraGSTs

In the present study, a total of 75* BraGST* genes were identified in* B. rapa*, through genome-wide identification. Based on the domain information and phylogenetic analysis of the GSTs, they were categorized into eight subclasses in which Tau subclass was the most numerous with 37 number of genes and Phi with 22. Our analysis was further validated from previous findings with dominant number of genes and the variation of copy number in plants [36, 49–52]. However, other subclasses have contained smaller number of genes with 1–4 members of GSTs. Furthermore, the results of our findings also showed a higher copy number of genes involved in the two subclasses Phi and Tau, which suggested that, after following whole genome duplication (WGD) event, it had a high degree of genes retention. As a result, our findings proved the gene dosage hypothesis for keeping network stability, following polyploidy, and underrepresented in copy variants the genes encoding members were preferentially retained [53, 54]. In the evolutionary process, gene duplications are necessary for new biological functions and widespread expansion of gene family [55]. To understand duplication events, here we analyzed the expansion mechanism of BraGSTs family by MCScanX program. A large number (54.67%) of segmental type duplications were identified compared to tandem (45.33%). These analyses suggested that segmental duplication contributed in the expansion of BraGSTs family. Moreover, gene duplications played a major role in diversification by altering the genetic setup to assist them in adaptation to environmental stresses [56]. Chinese cabbage showed more close evolutionary resemblance to model plant* A. thaliana* [57]. They also shared the identical genomic composition but their sizes differ from each other which is ~120 Mb in* Arabidopsis* [58, 59]. Meanwhile, genome of* B. rapa *exceeds to 529 Mb, which is five times larger than that of* Arabidopsis* [60, 61]. On the other hand, the modern diploid* Brassica* genome carried complexity among their three subgenomes, which are indicated in some of the preliminary studies [62–64]. In addition, we also found the divergence of GSTs with an average of 29.80 MYA, which has suggested a low selective pressure on BraGSTs that made them duplicate late, further demonstrated the contrasting nature with different function in subclasses of GSTs as discussed earlier. Moreover, on our results' basis, significant variation in rate of divergence reflected the similarities of* BraGST* sequences possibly matched with* A. thaliana* and rice [65, 66]. Additionally, all the selected tandem pairs showed lower than 1.00 synonymous values, which lie in purifying selection nature and help in the maintenance of* BraGST* gene functions. The results were further validated by the synonymous values which did not show any significant differences among three subgenomes of* B. rapa* (LF, MF1, and MF2). The comparative analysis, phylogenetic tree, and gene structure of GSTs revealed the exon-intron accordingly to their subclasses. The similarities in genes structure and the similar patterns of exon-intron were further proved by analyzing the protein highly conserved sequences with MEME. The regulation patterns for conserved motif were tight specifically linked with motifs 1 and 2, as these were determined the most prominent in the BraGSTs family.

### 4.2. Expression Divergence and Regulatory Network of BraGSTs

To validate our results, we also identified cis-regulatory elements in the promoter regions of BraGSTs specific to hormonal stress. We observed that the majority of GSTs were involved in the activity of methyl jasmonate. Therefore, we can speculate that the function of* BraGST *genes was involved in the regulatory phenomena of hormones. In addition, the expansion of this larger gene family and the duplicated genes in both models (neofunctionalization or subfunctionalization) was more associated with process of tissue-expression divergence [67–69]. We also examined the tissue-expression patterns of the* BraGST* genes; most of them were expressed across five tissues or several at least. Three genes were tissue-specific (two in roots and one in silique) while some of them showed similar patterns. That might suggested their common importance in the regulation of plant developments. The tissue-specific genes along with the tandem pairs might be contributing to plant development by acquiring new functions. Based on the results of RNA-seq data for various tissues and the response of prompter sequence analysis for cis-elements, their involvement in* B. rapa* response to different stresses was speculated. Thus, an opportunity to study the expression profile of* BraGST* genes was provided as to understand gene functions; the expression profiling can provide valuable clues [70]. Furthermore, the expression profile for 15 paralogous pairs was analyzed by qRT-PCR after application of eight multiple hormonal treatments (namely, ABA, GA, BR, ETH, IAA, IBA, NPA, and JA). In general, most of the plant growth hormones act as components of abiotic stress signaling, for instance, ABA, GA, Auxin, BR, cytokinin, and JA [71]. A large variety of cellular processes are regulated by phytohormones, although these are produced in minute concertation. To communicate cellular activities in higher plants, they work as chemical messengers [72]. The ratio for majority of treatments was upregulated in our study, such as for NPA stress (66%) and BR (61%); however JA was more sensitive as a large number (74%) of genes were downregulated. These results show that BraGSTs may also function in response to multiple hormone treatments, although most of their genes functions are still unknown. However, our analysis for phylogenetics, cis-elements, and expression profiling provides a foundation for future studies on* BraGST* gene functions. Although, based on PCC values, BR and IBA stress were among the highest with 10 PCC values each (>0.6) which signify a close relationship. Here, we speculated that the function of gene was enhanced and expanded through gene duplications. During the process of evolution after duplications, the divergence in the expression profile of the tandem pairs revealed that it may acquire new functions; however functional analysis will confirm and determine the pivotal role of BraGSTs. To understand regulatory network, protein-protein interactions were elucidated and most of the genes presented a close relationship among subclasses, except for few genes. All the* BraGST* genes displayed a very complicated correlation, suggesting that these genes are involved in several fundamental mechanisms and are further regulated by many down-/upstream genes. Taken together our results, this study may provide novel insight into the unique features and specifically the role of the BraGSTs family in eukaryotic organisms.

## 5. Conclusion

In this study, we identified 75* BraGST* genes from* B. rapa* and focused on those involved in response to multiple hormonal treatments as GSTs are playing a crucial role in plants. The classification, phylogenetic relationship, structural composition, evolutionary characteristics, and conserved protein motif analyses were investigated. Our study has provided a deep understanding of the BraGSTs in* B. rapa.* The differential expression patterns of BraGSTs in various tissues and the visible tissue-specific patterns showed that these genes are playing a key role in the developmental aspects of* B. rapa*. Expression analysis highlighted the involvement of* BraGST* genes in response to multiple hormone treatments. Furthermore, a highly interacting network and the correlation among various treatments demonstrated the importance of our study. These results will lead to novel insight by facilitating into functional divergence and will provide an assessment for further studies to understand the physiological function of GSTs in response to multiple hormone treatments in* B. rapa*.

## Figures and Tables

**Table 1:** The *BraGST* genes of *Brassica rapa* were classified based on their domain and phylogenetic relationships.

Subfamilies	Identified genes
Phi	22
Tau	37
Zeta	3
Theta	2
Lambda	3
DHAR	4
EF1G	3
TCHQD	1

Total	75

**Table 2:** *Ka*/*Ks* calculation of the paralog pairs (tandem array) of BraGSTs in *B. rapa*.

Genes	*Ks*	*Ka*	*Ka*/*Ks*	Selection pressure	Duplication time (MYA)
BraGSTF16_BraGSTF2	1.89	0.34	0.18	Tandem array	63.13
BraGSTF13_BraGSTF22	0.14	0.11	0.77	Tandem array	4.74
BraGSTF4_BraGSTF5	0.23	0.06	0.27	Tandem array	7.81
BraGSTF6_BraGSTF7	0.12	0.08	0.68	Tandem array	3.89
BraGSTF19_BraGSTF20	1.18	0.47	0.39	Tandem array	39.44
BraGSTU6_BraGSTU3	1.07	0.33	0.31	Tandem array	35.78
BraGSTU4_BraGSTU5	0.66	0.15	0.22	Tandem array	21.83
BraGSTU2_BraGSTU8	0.80	0.24	0.30	Tandem array	26.57
BraGSTU11_BraGSTU23	0.64	0.15	0.24	Tandem array	21.26
BraGSTU12_BraGSTU14	0.26	0.10	0.39	Tandem array	8.77
BraGSTU13_BraGSTU20	0.99	0.16	0.16	Tandem array	33.00
BraGSTU21_BraGSTU34	1.45	0.26	0.18	Tandem array	48.17
BraGSTU26_BraGSTU32	1.01	0.24	0.24	Tandem array	33.63
BraGSTU27_BraGSTU28	0.72	0.11	0.16	Tandem array	23.90
BraGSTU30_BraGSTU33	2.25	0.30	0.13	Tandem array	75.10

## Supplementary Materials

Supplementary Materials Table 1: Sequences of the* BraGST* gene primers used for quantitative real-time PCR. Table 2: The basic description of* BraGST* genes in* Brassica rapa*. Table 3: Identification of BraGST syntenic genes between* A. thaliana* along with three subgenomes of* B. rapa*. Table 4: The FPKM values of* BraGST* genes. Table 5: Syntenic paralog pairs of BraGSTs with PC and FPKM values. Table 6: Cis-elements of* BraGST* genes in* Brassica rapa*. Table 7: Relative expression pattern of* BraGST* genes along with PC values with respect to hormonal stresses by qRT-PCR. Table 8: Pearson correlation coefficient of the stress-induced* BraGSTs* whose PC is greater than 0.5. Figure S1: Genetic distance among different subclasses of BraGSTs. Figure S2: Genetic distance among different subclasses of BraGSTs. Figure S3: Relative shares of different family among three subgenomes of* B. rapa*. Figure S4: Evolutionary tree of BraGSTs and the different subclasses being displayed with random color and tree generated with MEGA 7 using 1000-bootstrap replicate values.

## References

[1] Dixon D. P., Skipsey M., Edwards R. (2010). Roles for glutathione transferases in plant secondary metabolism. *Phytochemistry*.

[2] Cummins I., Dixon D. P., Freitag-Pohl S., Skipsey M., Edwards R. (2011). Multiple roles for plant glutathione transferases in xenobiotic detoxification. *Drug Metabolism Reviews*.

[3] Marrs K. A., Alfenito M. R., Lloyd A. M., Walbot V. (1995). A glutathione S-transferase involved in vacuolar transfer encoded by the maize gene Bronze-2. *Nature*.

[4] Alfenito M. R., Souer E., Goodman C. D. (1998). Functional complementation of anthocyanin sequestration in the vacuole by widely divergent glutathione S-transferases. *The Plant Cell*.

[5] Kitamura S., Shikazono N., Tanaka A. (2004). TRANSPARENT TESTA 19 is involved in the accumulation of both anthocyanins and proanthocyanidins in Arabidopsis. *The Plant Journal*.

[6] Kitamura S., Akita Y., Ishizaka H., Narumi I., Tanaka A. (2012). Molecular characterization of an anthocyanin-related glutathione S-transferase gene in cyclamen. *Journal of Plant Physiology*.

[7] Chi Y., Cheng Y., Vanitha J. (2011). Expansion mechanisms and functional divergence of the glutathione S-transferase family in sorghum and other higher plants. *DNA Research*.

[8] Csiszár J., Horváth E., Váry Z. (2014). Glutathione transferase supergene family in tomato: Salt stress-regulated expression of representative genes from distinct GST classes in plants primed with salicylic acid. *Plant Physiology and Biochemistry*.

[9] Labrou N. E., Papageorgiou A. C., Pavli O., Flemetakis E. (2015). Plant GSTome: Structure and functional role in xenome network and plant stress response. *Current Opinion in Biotechnology*.

[10] Axarli I., Dhavala P., Papageorgiou A. C., Labrou N. E. (2009). Crystallographic and Functional Characterization of the Fluorodifen-inducible Glutathione Transferase from Glycine max Reveals an Active Site Topography Suited for Diphenylether Herbicides and a Novel L-site. *Journal of Molecular Biology*.

[11] Axarli I., Dhavala P., Papageorgiou A. C., Labrou N. E. (2009). Crystal structure of glycine max glutathione transferase in complex with glutathione: Investigation of the mechanism operating by the Tau class glutathione transferases. *Biochemical Journal*.

[12] Frova C. (2006). Glutathione transferases in the genomics era: New insights and perspectives. *Biomolecular Engineering*.

[13] Frova C. (2003). The plant glutathione transferase gene family: genomic structure, functions, expression and evolution. *Physiologia Plantarum*.

[14] Karavangeli M., Labrou N. E., Clonis Y. D., Tsaftaris A. (2005). Development of transgenic tobacco plants overexpressing maize glutathione S-transferase I for chloroacetanilide herbicides phytoremediation. *Biomolecular Engineering*.

[15] Sharma R., Sahoo A., Devendran R., Jain M. (2014). Over-expression of a rice tau class glutathione S-transferase gene improves tolerance to salinity and oxidative stresses in arabidopsis. *PLoS ONE*.

[16] Tiwari V., Patel M. K., Chaturvedi A. K., Mishra A., Jha B. (2016). Functional characterization of the tau class glutathione-S-transferases gene (SbGSTU) promoter of Salicornia brachiata under salinity and osmotic stress. *PLoS ONE*.

[17] Roxas V. P., Smith R. K., Allen E. R., Allen R. D. (1997). Overexpression of glutathione S-transferase/glutathione peroxidase enhances the growth of transgenic tobacco seedlings during stress. *Nature Biotechnology*.

[18] Dixon D. P., Davis B. G., Edwards R. (2002). Functional divergence in the glutathione transferase superfamily in plants: Identification of two classes with putative functions in redox homeostasis in Arabidopsis thaliana. *The Journal of Biological Chemistry*.

[19] Dixon D. P., Edwards R. (2010). Roles for stress-inducible lambda glutathione transferases in flavonoid metabolism in plants as identified by ligand fishing. *The Journal of Biological Chemistry*.

[20] Kwon S.-Y., Choi S.-M., Ahn Y.-O. (2003). Enhanced stress-tolerance of transgenic tobacco plants expressing a human dehydroascorbate reductase gene. *Journal of Plant Physiology*.

[21] Chen Z., Gallie D. R. (2006). Dehydroascorbate reductase affects leaf growth, development, and function. *Plant Physiology*.

[22] Ushimaru T., Nakagawa T., Fujioka Y. (2006). Transgenic Arabidopsis plants expressing the rice dehydroascorbate reductase gene are resistant to salt stress. *Journal of Plant Physiology*.

[23] Thom R., Dixon D. P., Edwards R., Cole D. J., Lapthorn A. J. (2001). The structure of a zeta class glutathione S-transferase from Arabidopsis thaliana: Characterisation of a GST with novel active-site architecture and a putative role in tyrosine catabolism. *Journal of Molecular Biology*.

[24] Basantani M., Srivastava A. (2007). Plant glutathione transferases - A decade falls short. *Botany*.

[25] Vickers T. J., Wyllie S., Fairlamb A. H. (2004). Leishmania major elongation factor 1B complex has trypanothione S-transferase and peroxidase activity. *The Journal of Biological Chemistry*.

[26] Hasanuzzaman M., Nahar K., Anee T. I., Fujita M. (2017). Glutathione in plants: biosynthesis and physiological role in environmental stress tolerance. *Physiology and Molecular Biology of Plants*.

[27] Zmorzyński S., Świderska-Kołacz G., Koczkodaj D., Filip A. A. (2015). Significance of Polymorphisms and Expression of Enzyme-Encoding Genes Related to Glutathione in Hematopoietic Cancers and Solid Tumors. *BioMed Research International*.

[28] Aquilano K., Baldelli S., Ciriolo M. R. (2014). Glutathione: new roles in redox signaling for an old antioxidant. *Frontiers in Pharmacology*.

[29] Marí M., Morales A., Colell A., García-Ruiz C., Fernández-Checa J. C. (2009). Mitochondrial glutathione, a key survival antioxidant. *Antioxidants & Redox Signaling*.

[30] Marí M., Colell A., Morales A., Von Montfort C., Garcia-Ruiz C., Fernández-Checa J. C. (2010). Redox control of liver function in health and disease. *Antioxidants & Redox Signaling*.

[31] Øverby A., Stokland R. A., Åsberg S. E., Sporsheim B., Bones A. M. (2015). Allyl isothiocyanate depletes glutathione and upregulates expression of glutathione S-transferases in Arabidopsis thaliana. *Frontiers in Plant Science*.

[32] Tong C., Wang X., Yu J. (2013). Comprehensive analysis of RNA-seq data reveals the complexity of the transcriptome in Brassica rapa. *BMC Genomics*.

[33] Town C. D., Cheung F., Maiti R. (2006). Comparative genomics of Brassica oleracea and Arabidopsis thaliana reveal gene loss, fragmentation, and dispersal after polyploidy. *The Plant Cell*.

[34] Wang X., Wang H., Wang J. (2011). The genome of the mesopolyploid crop species Brassica rapa. *Nature Genetics*.

[35] Kawahara Y., de la Bastide M., Hamilton J. P. (2013). Improvement of the *Oryza sativa* Nipponbare reference genome using next generation sequence and optical map data. *Rice*.

[36] Jain M., Ghanashyam C., Bhattacharjee A. (2010). Comprehensive expression analysis suggests overlapping and specific roles of rice glutathione S-transferase genes during development and stress responses. *BMC Genomics*.

[37] Edgar R. C. (2004). MUSCLE: multiple sequence alignment with high accuracy and high throughput. *Nucleic Acids Research*.

[38] Kumar S., Stecher G., Tamura K. (2016). MEGA7: Molecular Evolutionary Genetics Analysis version 7.0 for bigger datasets. *Molecular Biology and Evolution*.

[39] Bailey T. L., Boden M., Buske F. A. (2009). MEME SUITE: tools for motif discovery and searching. *Nucleic Acids Research*.

[40] Koch M. A., Haubold B., Mitchell-Olds T. (2000). Comparative evolutionary analysis of chalcone synthase and alcohol dehydrogenase loci in Arabidopsis, Arabis, and related genera (Brassicaceae). *Molecular Biology and Evolution*.

[41] Voorrips R. E. (2002). Mapchart: software for the graphical presentation of linkage maps and QTLs. *Journal of Heredity*.

[42] Cheng F., Wu J., Fang L., Wang X. (2012). Syntenic gene analysis between Brassica rapa and other Brassicaceae species. *Frontiers in Plant Science*.

[43] Krzywinski M., Schein J., Birol I. (2009). Circos: An information aesthetic for comparative genomics. *Genome Research*.

[44] Heid C. A., Stevens J., Livak K. J., Williams P. M. (1996). Real time quantitative PCR. *Genome Research*.

[45] Dixon D. P., Lapthorn A., Edwards R. (2002). Plant glutathione transferases. *Genome Biology*.

[46] Edwards R., Dixon D. P., Walbot V. (2000). Plant glutathione *S*-transferases: enzymes with multiple functions in sickness and in health. *Trends in Plant Science*.

[47] Sappl P. G., Carroll A. J., Clifton R. (2009). The *Arabidopsis* glutathione transferase gene family displays complex stress regulation and co-silencing multiple genes results in altered metabolic sensitivity to oxidative stress. *The Plant Journal*.

[48] Dixon D. P., Cummins L., Cole D. J., Edwards R. (1998). Glutathione-mediated detoxification systems in plants. *Current Opinion in Plant Biology*.

[49] Lan T., Wang X.-R., Zeng Q.-Y. (2013). Structural and functional evolution of positively selected sites in pine glutathione S-transferase enzyme family. *The Journal of Biological Chemistry*.

[50] Lan T., Yang Z.-L., Yang X., Liu Y.-J., Wang X.-R., Zenga Q.-Y. (2009). Extensive functional diversification of the populus glutathione s-transferase supergene family. *The Plant Cell*.

[51] Liu H.-J., Tang Z.-X., Han X.-M. (2015). Divergence in enzymatic activities in the soybean GST supergene family provides new insight into the evolutionary dynamics of whole-genome duplicates. *Molecular Biology and Evolution*.

[52] Liu Y.-J., Han X.-M., Ren L.-L., Yang H.-L., Zeng Q.-Y. (2013). Functional divergence of the glutathione S-transferase supergene family in physcomitrella patens reveals complex patterns of large gene family evolution in land plants. *Plant Physiology*.

[53] Birchler J. A., Veitia R. A. (2007). The gene balance hypothesis: from classical genetics to modern genomics. *The Plant Cell*.

[54] Lou P., Wu J., Cheng F., Cressman L. G., Wang X., Robertson McClung C. (2012). Preferential retention of circadian clock genes during diploidization following whole genome triplication in Brassica rapa. *The Plant Cell*.

[55] Hurles M. (2004). Gene duplication: The genomic trade in spare parts. *PLoS Biology*.

[56] Taylor J. S., Raes J. (2004). Duplication and divergence: The evolution of new genes and old ideas. *Annual Review of Genetics*.

[57] The Brassica rapa Genome Sequencing Project C, Wang X., Wang H., Wang J. (2011). The genome of the mesopolyploid crop species *Brassica rapa*. *Nature Genetics*.

[58] Arabidopsis I. G. (2000). Analysis of the genome sequence of the flowering plant *Arabidopsis thaliana*. *Nature*.

[59] Bennett M. D., Leitch I. J., Price H. J., Johnston J. S. (2003). Comparisons with Caenorhabditis (~100 Mb) and Drosophila (~175 Mb) using flow cytometry show genome size in Arabidopsis to be ~157 Mb and thus ~25% larger than the Arabidopsis genome initiative estimate of ~125 Mb. *Annals of Botany*.

[60] Johnston J. S., Pepper A. E., Hall A. E. (2005). Evolution of genome size in Brassicaceae. *Annals of Botany*.

[61] Lysak M. A., Koch M. A., Beaulieu J. M., Meister A., Leitch I. J. (2009). The dynamic ups and downs of genome size evolution in Brassicaceae. *Molecular Biology and Evolution*.

[62] Lagercrantz U., Putterill J., Coupland G., Lydiate D. (1996). Comparative mapping in *Arabidopsis* and *Brassica*, fine scale genome collinearity and congruence of genes controlling flowering time. *The Plant Journal*.

[63] Scheffler J. A., Sharpe A. G., Schmidt H. (1997). Desaturase multigene families of Brassica napus arose through genome duplication. *Theoretical and Applied Genetics*.

[64] Cavell A. C., Lydiate D. J., Parkin I. A. P., Dean C., Trick M. (1998). Collinearity between a 30-centimorgan segment of Arabidopsis thaliana chromosome 4 and duplicated regions within the Brassica napus genome. *Genome*.

[65] Simillion C., Vandepoele K., Van Montagu M. C. E., Zabeau M., Van de Peer Y. (2002). The hidden duplication past of Arabidopsis thaliana. *Proceedings of the National Acadamy of Sciences of the United States of America*.

[66] Yim W. C., Lee B.-M., Jang C. S. (2009). Expression diversity and evolutionary dynamics of rice duplicate genes. *Molecular Genetics and Genomics*.

[67] Ganko E. W., Meyers B. C., Vision T. J. (2007). Divergence in expression between duplicated genes in Arabidopsis. *Molecular Biology and Evolution*.

[68] Li W.-H., Yang J., Gu X. (2005). Expression divergence between duplicate genes. *Trends in Genetics*.

[69] Huerta-Cepas J., Dopazo J., Huynen M. A., Gabaldón T. (2011). Evidence for short-time divergence and long-time conservation of tissue-specific expression after gene duplication. *Briefings in Bioinformatics*.

[70] Ma H., Zhao J. (2010). Genome-wide identification, classification, and expression analysis of the arabinogalactan protein gene family in rice (Oryza sativa L.). *Journal of Experimental Botany*.

[71] Wani S. H., Kumar V., Shriram V., Sah S. K. (2016). Phytohormones and their metabolic engineering for abiotic stress tolerance in crop plants. *Crop Journal*.

[72] Voß U., Bishopp A., Farcot E., Bennett M. J. (2014). Modelling hormonal response and development. *Trends in Plant Science*.

