# Supplementary figures and images for: Genome-Wide Identification, Classification, and Expression Divergence of Glutathione-Transferase Family in Brassica rapa under Multiple Hormone Treatments
Source: Biomed Res Int. 2018 May 24;2018:6023457. doi: 10.1155/2018/6023457 (PMC5994329; doi:10.1155/2018/6023457)

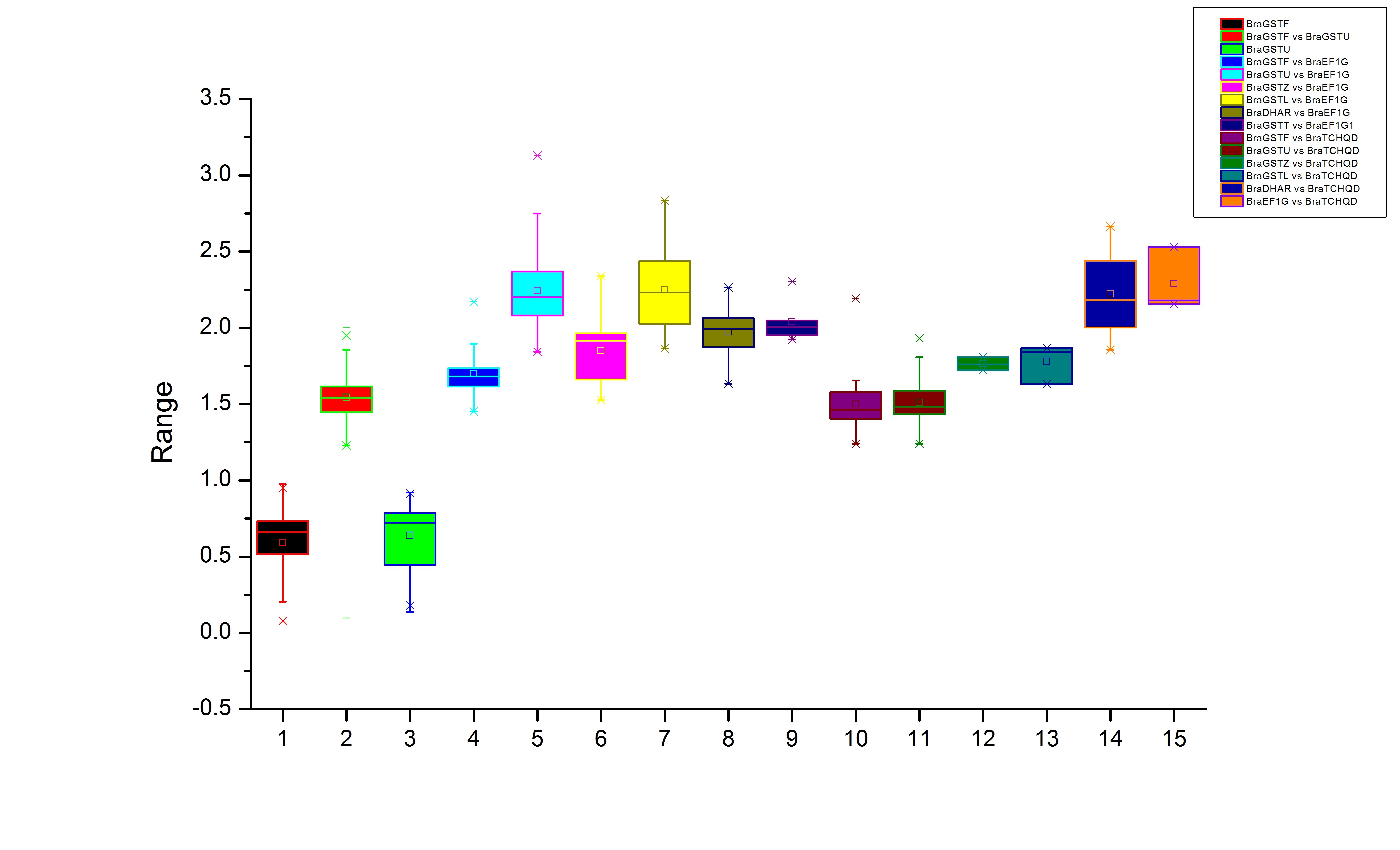

Supplement: Supplementary Materials — Table 1: Sequences of the BraGST gene primers used for quantitative real-time PCR. Table 2: The basic description of BraGST genes in Brassica rapa. Table 3: Identification of BraGST syntenic genes between A. thaliana along with three subgenomes of B. rapa. Table 4: The FPKM values of BraGST genes. Table 5: Syntenic paralog pairs of BraGSTs with PC and FPKM values. Table 6: Cis-elements of BraGST genes in Brassica rapa. Table 7: Relative expression pattern of BraGST genes along with PC values with respect to hormonal stresses by qRT-PCR. Table 8: Pearson correlation coefficient of the stress-induced BraGSTs whose PC is greater than 0.5. Figure S1: Genetic distance among different subclasses of BraGSTs. Figure S2: Genetic distance among different subclasses of BraGSTs. Figure S3: Relative shares of different family among three subgenomes of B. rapa. Figure S4: Evolutionary tree of BraGSTs and the different subclasses being displayed with random color and tree generated with MEGA 7 using 1000-bootstrap replicate values. [file 6023457.f1.zip › Fig. S1.jpg]

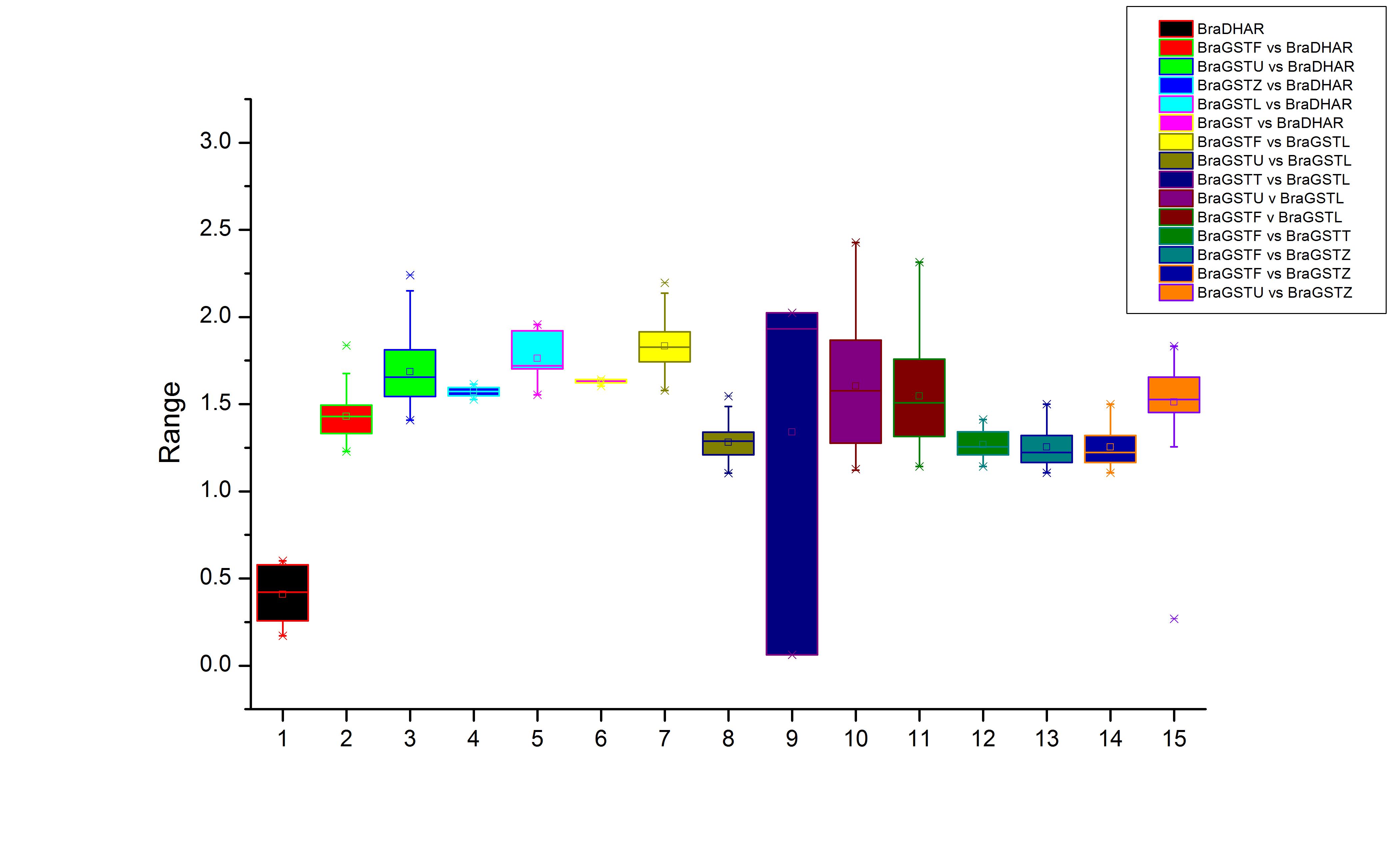

Supplement: Supplementary Materials — Table 1: Sequences of the BraGST gene primers used for quantitative real-time PCR. Table 2: The basic description of BraGST genes in Brassica rapa. Table 3: Identification of BraGST syntenic genes between A. thaliana along with three subgenomes of B. rapa. Table 4: The FPKM values of BraGST genes. Table 5: Syntenic paralog pairs of BraGSTs with PC and FPKM values. Table 6: Cis-elements of BraGST genes in Brassica rapa. Table 7: Relative expression pattern of BraGST genes along with PC values with respect to hormonal stresses by qRT-PCR. Table 8: Pearson correlation coefficient of the stress-induced BraGSTs whose PC is greater than 0.5. Figure S1: Genetic distance among different subclasses of BraGSTs. Figure S2: Genetic distance among different subclasses of BraGSTs. Figure S3: Relative shares of different family among three subgenomes of B. rapa. Figure S4: Evolutionary tree of BraGSTs and the different subclasses being displayed with random color and tree generated with MEGA 7 using 1000-bootstrap replicate values. [file 6023457.f1.zip › Fig. S2.jpg]

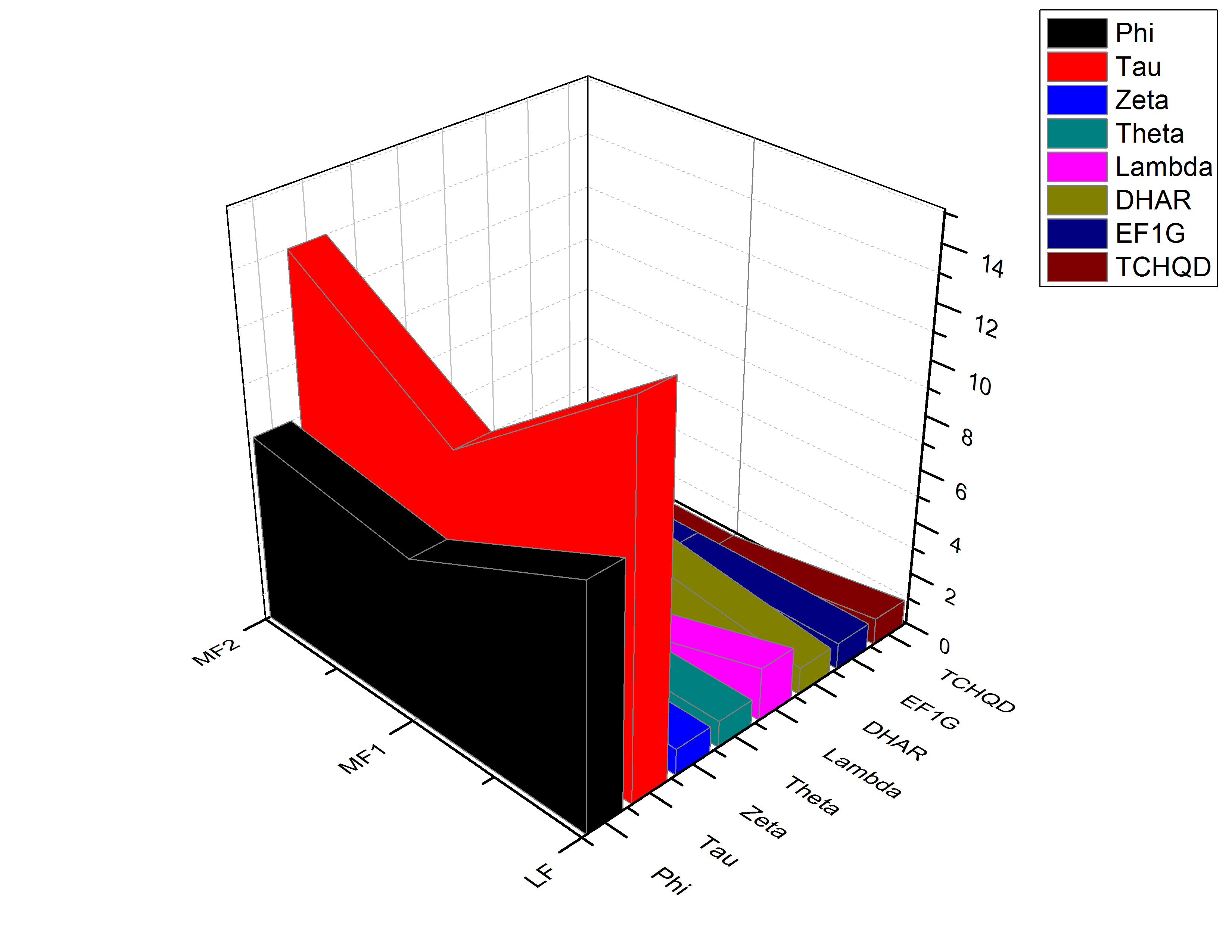

Supplement: Supplementary Materials — Table 1: Sequences of the BraGST gene primers used for quantitative real-time PCR. Table 2: The basic description of BraGST genes in Brassica rapa. Table 3: Identification of BraGST syntenic genes between A. thaliana along with three subgenomes of B. rapa. Table 4: The FPKM values of BraGST genes. Table 5: Syntenic paralog pairs of BraGSTs with PC and FPKM values. Table 6: Cis-elements of BraGST genes in Brassica rapa. Table 7: Relative expression pattern of BraGST genes along with PC values with respect to hormonal stresses by qRT-PCR. Table 8: Pearson correlation coefficient of the stress-induced BraGSTs whose PC is greater than 0.5. Figure S1: Genetic distance among different subclasses of BraGSTs. Figure S2: Genetic distance among different subclasses of BraGSTs. Figure S3: Relative shares of different family among three subgenomes of B. rapa. Figure S4: Evolutionary tree of BraGSTs and the different subclasses being displayed with random color and tree generated with MEGA 7 using 1000-bootstrap replicate values. [file 6023457.f1.zip › Fig. S3.jpg]

**Tau**  
**Lambda**  
**EF1G**  
**Zeta**  
**Theta**  
**TCHQD**  
**Phi**

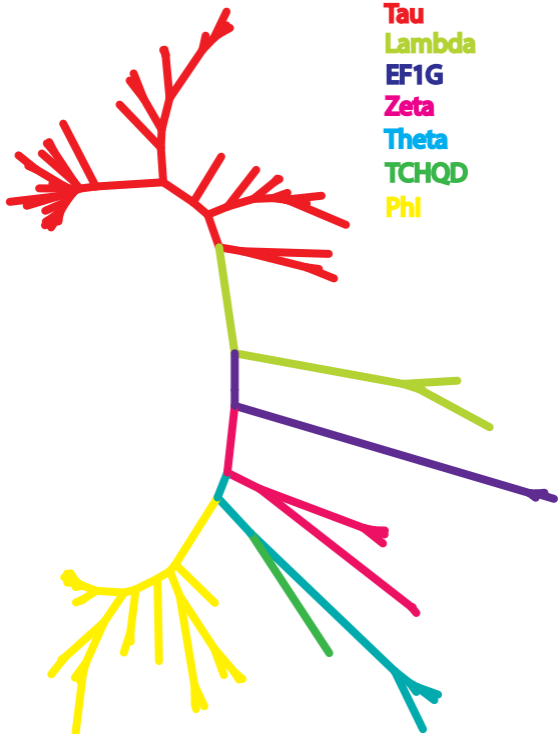

0.2

Supplement: Supplementary Materials — Table 1: Sequences of the BraGST gene primers used for quantitative real-time PCR. Table 2: The basic description of BraGST genes in Brassica rapa. Table 3: Identification of BraGST syntenic genes between A. thaliana along with three subgenomes of B. rapa. Table 4: The FPKM values of BraGST genes. Table 5: Syntenic paralog pairs of BraGSTs with PC and FPKM values. Table 6: Cis-elements of BraGST genes in Brassica rapa. Table 7: Relative expression pattern of BraGST genes along with PC values with respect to hormonal stresses by qRT-PCR. Table 8: Pearson correlation coefficient of the stress-induced BraGSTs whose PC is greater than 0.5. Figure S1: Genetic distance among different subclasses of BraGSTs. Figure S2: Genetic distance among different subclasses of BraGSTs. Figure S3: Relative shares of different family among three subgenomes of B. rapa. Figure S4: Evolutionary tree of BraGSTs and the different subclasses being displayed with random color and tree generated with MEGA 7 using 1000-bootstrap replicate values. [file 6023457.f1.zip › Fig. S4.pdf]
